# Evaluation of competence training for the minimally trained health worker in type 2 diabetes: A cluster randomized controlled trial

**DOI:** 10.1097/MD.0000000000022959

**Published:** 2020-10-30

**Authors:** Anirudh Gaurang Gudlavalleti, Giridhara R. Babu, Onno C.P. van Schayck, Nicolaas C. Schaper, Melissa Glenda Lewis, G.V.S. Murthy

**Affiliations:** a Indian Institute of Public Health Hyderabad, Public Health Foundation of India, ANV Arcade, 1 Amar Cooperative Society, Kavuri Hills, Madhapur, Hyderabad, India –; b CAPHRI CaRE and Public Health Research Institute, Maastricht University, PO Box 616, 6200 MD Maastricht, the Netherlands.

**Keywords:** diabetes, diabetes management, diabetes training, health systems, health worker, health worker training, India

## Abstract

**Introduction:**

Type 2 diabetes mellitus is responsible for high mortality and morbidity globally and in India. India has high prevalence of the condition and the burden is set to increase exponentially in the next decade. Indians traditionally reside in rural or semi-urban areas with limited access to healthcare facilities. To overcome this, the government has introduced a cadre of health workers called Accredited Social Health Activists (ASHA) for such areas. These workers were initially trained to provide maternal & infant care but now need improved competence training to improve type 2 diabetes screening & management in these locations. The objective of the study is to assess the competence training provided to ASHA workers at the chosen study sites.

**Methodology:**

A cluster randomized control trial has been designed. It will be conducted across 8 centers in Hyderabad & Rangareddy districts of Telangana, India. The training will be provided to ASHA workers. The tool used for training will be developed from existing sources with an emphasis on topics which require training. The training will be delivered across 6 months at each center as a classroom training. Each participant's baseline competence will be recorded using a questionnaire tool and a practical evaluation by trained public health experts. The same experts will use the same tools to assess the training post the intervention.

**Discussion:**

This trial will evaluate the use of health worker training as a tool for improving the clinical competence in relation to type 2 diabetes mellitus. We anticipate that the module will provide a greater understanding of type 2 diabetes mellitus, the importance of screening of both disease and complications and improved skills for the same. The study has received the ethical approval form the Institutional Ethics Committee of the Indian Institute of Public Health Hyderabad. The registration number is: IIPHH/TRCIEC/218/2020. The trial has also been registered under the Clinical trial registry of India (CTRI) on 27^th^ July 2020. The registration number of the trial is: CTRI/2020/07/026828. The URL of the registry trial is: http://ctri.nic.in/Clinicaltrials/showallp.php?mid1=45342&EncHid=&userName=CTRI/2020/07/026828.

## Abstract 2

Supplemental Digital Content is available in the text

## 1 Introduction

Type 2 diabetes mellitus results in high mortality and morbidity globally.^[1]^ The International Diabetes Federation estimates 75 million persons living with diabetes mellitus in India. Amongst them, 1.1 million adults (aged 20–79 years) die annually due to type 2 diabetes mellitus.^[2]^ Estimates also indicate around 42.2 million Indians currently living with undiagnosed type 2 diabetes mellitus.^[2]^ The total type 2 diabetes mellitus disorder burden in India is enormous, and the burden is set to increase if not managed. Hence, timely diagnosis/screening and management need to be scaled up in the country. The Indian Council of Medical Research has set forth guidelines for screening and management of type 2 diabetes.^[3]^

Most Indians (66.4% in 2017) live in the rural and semi-urban areas.^[4]^ They have limited access to healthcare facilities due to socio-economic status, distance, availability of facilities, inadequate number of skilled doctors, etc..^[5–10]^ Concurrently, the prevalence of type 2 diabetes mellitus is increasing in rural and semi-urban India. More individuals from the lower socio-economic status are now being identified with type 2 diabetes mellitus.^[11]^ Such persons with diabetes mellitus face catastrophic consequences. This is due to increasing type 2 diabetes mellitus burden and poor accessibility to public healthcare facilities. Hence, an innovative and feasible strategy is needed to improve access for efficient type 2 diabetes mellitus screening and management.

The use of minimally trained health workers in improving access to healthcare facilities has proven successful in low and middle-income countries.^[12–14]^ The successes have been observed for communicable and non-communicable diseases.^[15–26]^ Hence, type 2 diabetes mellitus screening and management by health workers in India offers a potentially feasible strategy. It can improve screening and subsequent management of type 2 diabetes mellitus and associated complications at the community level.

The Indian government in 2005, created a new cadre of non-physician minimally trained health volunteers. They are called accredited social health activists. These activists are female health volunteers. They are the primary interface between the public health care facilities and rural communities. They handle social mobilization and awareness in the 1000 residents of their community. Their primary activities include mobilizing women and children for reproductive and child health services, immunization of newborn, accompanying the referred cases for complications, etc. They are paid case-based incentives for her services.^[5,17]^ Currently, the government is also looking at utilizing the accredited social health activists’ services for non-communicable disease management.^[27]^ In lieu of the same, the government's National Program for prevention and control of Cancer, Diabetes, Cardiovascular diseases and Stroke has also formulated operational guidelines for the health workers^[28]^

Competence has been explained as the “*possession of requisite skills, knowledge, education and capacity*”.^[29]^ The competence about type 2 diabetes mellitus and its complications is a limiting factor in utilizing this workforce. It is important to provide standardized training to the health workers and then utilize their services in type 2 diabetes mellitus.^[18]^ Ultimately the training should improve the competency of the health workers to improve type 2 diabetes mellitus screening at the public healthcare facilities.

### 1.1 Aim and objectives of the study

#### 1.1.1 Aim

We aim to assess the effect of health worker training in improving their competence with respect to type 2 diabetes mellitus.

#### 1.1.2 Objectives of the study

The following are the objectives:

a. To train the health workers in competency-based training for type 2 diabetes mellitus.
b. To evaluate the training in realizing the change in the competence of the health workers.

### 1.2 Primary hypothesis

We hypothesize that the competence levels of the health workers in the intervention package will increase the competence levels to 60% compared to an estimated level of 30% at the baseline.

## 2 Methods

### 2.1.1 Preliminary phase

This phase involves, identification of the study areas, the needs assessment with important stakeholders in these study areas and the designing and validation of the training module.

The training module will be collated using the modules developed by the Ministry of Health and Family Welfare, Government of India, The Indian Council of Medical Research, The National Program for Prevention and Control of Cancer, Diabetes, Cardiovascular diseases and Stroke, The Indian Institute of Public Health-Hyderabad and the Public Health Foundation of India will be utilized.^[3,30–32]^

Subsequently, a customized tool (questionnaire) will be developed for the evaluation of the competence, which will have both theoretical and practical components.

- *Identification of Study area:* Eight health centers across Hyderabad & Ranga Reddy districts of the state of Telangana will be identified as the study sites. Caution will be exercised to ensure that the eight centers are spread widely across the district. It will ensure no contamination of health workers and their patients from the intervention to the control group or vice-versa. These centers will also be identified based on their consent and the availability of health workers
- *Needs Assessment:* A needs assessment exercise will be conducted involving the important stakeholders involved. The stakeholders will consist of government officials, the medical officer at the health center and the health workers from the cadre of workers to be trained.
- *Designing and Validation of Intervention package (training and evaluation tools):* The training module and evaluation questionnaires developed will be used for validating the entire intervention package at a neutral site with 10% of the calculated sample size, that is, 30 health workers. The questionnaire will be developed to assess the change in competence pertaining to type 2 diabetes mellitus and its associated complications after the training. Pre and post-test assessment using the questionnaire and practical tools will be conducted in the test population for validating the training and evaluation tool.

### 2.2 Intervention phase

This phase will mainly consist of clustering of the health centers randomly into the intervention and control clusters, the identification and recruitment of health workers into the study based on the inclusion and exclusion criteria and the data collection procedure for training evaluation.

- *Clustering of health centers:* This stage will entail the random division of the identified eight health centers into two clusters namely the intervention cluster and the control cluster.
- *Identification & Recruitment of Health Workers:* In this stage after the health centers have been randomized into the intervention and control clusters, the health workers will be screened for eligibility. Those eligible will be inducted into the study based on their consent. The selection will be dependent on the inclusion and exclusion criteria mentioned under study design. The participants will be explained about the process using a participant information sheet. (See document- Appendix 1 Supplemental Digital Content 1 (Appendix 1), which illustrates the Participant Information sheet). This sheet will be shared with each participant after translating it into their vernacular language.
- *Data Collection:* Using a baseline questionnaire, we will understand their competence in type 2 diabetes mellitus. This tool will be developed using Michigan University's Diabetes Knowledge Test-2,^[33,34]^ evaluation tools developed by the government of India and PHFI along with inputs from endocrinologists/physicians/public health experts. This will help us assess the baseline competence levels of the health workers. This tool will also capture the baseline data for the clinical competence of the participating health workers. All socio-demographic details and baseline details will be collected, entered and analyzed later.

### 2.3 Study design

We will conduct a 12 months prospective, unblinded, parallel cluster randomized controlled trial. The study will measure changes in healthcare worker competence levels related to type 2 diabetes mellitus and complications. The health centers chosen as the clusters will be then randomized into intervention and control arms. The health workers at the respective health center will be thus assigned to the respective cluster arm. The clusters will be chosen such that they are geographically distinct. This will avoid any possible contamination within the groups. Health workers in the control group will continue to deliver services according to the current standards and guidelines.^[28,30,31,35]^ The intervention group participants will undergo additional training. This will be spread over six months and delivered for 20 hours.^[36]^ It will entail pre and post-assessment questionnaires and practical assessments as the modality of evaluation.

#### 2.3.1 Population, sample, and setting

Our target population will be health workers working in chosen health centers of Hyderabad & Rangareddy districts of Telangana. The requisite number of health workers (See document- Appendix 2, Supplemental Digital Content (Appendix 2), which illustrates the probable sites of study) will be chosen upon satisfying the inclusion criteria mentioned later.

#### 2.3.2 Sample size calculations

The sample size is calculated based on the following formulae: 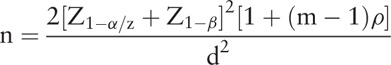


Where

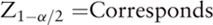
 to two-tailed at 5% significance level = 1.96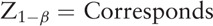 to 80% power = 0.84
d = effect size (estimated) = 0.5 (medium effect based on Cohen's d)
m = cluster size = 30 (minimum number of health workers at one health center)
ρ = intracluster correlation coefficient (estimated) = 0.03

we get n as 117 HWs per group. Adjusting the same for a dropout rate of 20%, 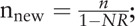 we get n as 147 per cluster group, i.e. 294 HWs across both arms. Therefore, 294 HWs across 8 clusters will be needed with 37 HWs across each cluster arm. Each cluster has 40–45 health workers and hence the sample size calculated is feasible for the study.

#### 2.3.3 Study participants

All health workers will be screened for eligibility based on the following inclusion criteria^[5,18,19]^:

i. Health workers employed as accredited social health activists
ii. Health workers living within a radius of 40kms from the identified health centers.

The exclusion criteria are:

i. Pregnant health workers
ii. Health workers unwilling/unable to provide written informed consent.
iii. Health workers with terminal illnesses.

#### 2.3.4 Sampling method

Cluster sampling method will be utilized for the study. First, 8 health centers will be chosen from the Hyderabad & Rangareddy districts and will further be then randomized as clusters into either the intervention or the control arms. All health workers at the respective clusters will be included in the respective arms based on their eligibility and consent.

A total of approximately 294 health workers will participate in the study, encouraged both by getting continuing education and by being urged by a district health officer to participate. We expect that approximately 147 health workers will enter the intervention arm, wherein they will participate in 20 hours of the intervention to be considered as trained. Evidence shows that even brief interventions have an impact on effective knowledge.^[36]^ The principal investigator and the field staff will take the requisite permission form authorities and enroll the participants into the study based on their consent, which will be recorded using a consent form.

**Figure 1:**
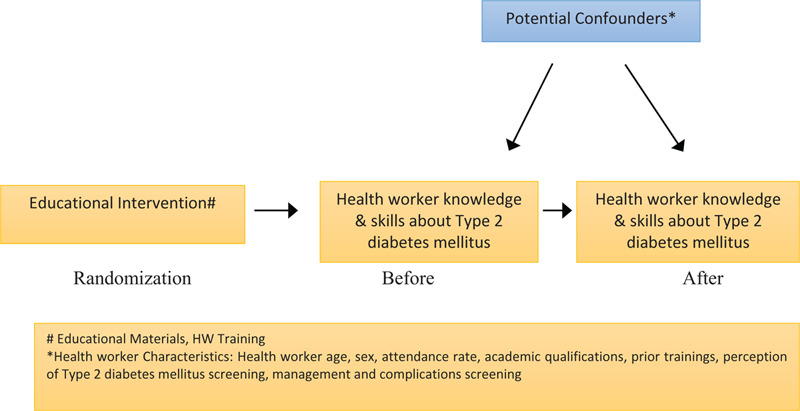
Primary Hypothesis displaying intervention, intermediate and outcome of interest.

To prevent possible confounding and effect modification of patients’ characteristics, we will divide the health centers into pairs. The pairs will have the following characteristics: male/female ratio, percentage of patients ≥ 60 years old, percentage of patients on treatment and the prevalence of patients suffering from chronic diseases. The centers will be then randomly allocated into an experimental group and a control group and will be compared to ascertain the balance of the allocation. This will be done using computer generated random number using the MS Excel software. Pre- and postassessment scores will be recorded at baseline and at after the intervention. These scores will serve as the data regarding competence assessment. Health workers’ characteristics will be not included in the randomization process. This data is not available prior to the information from the baseline questionnaire being received (Fig. 1 emphasizes the relationship, being examined in the evaluation). The intervention is randomized and is hence an important variable between exposure and outcome in the experimental group as well as the evaluation method (e.g., before and after study). Concurrently potential confounders will also be identified and would majorly consist of health workers’ characteristics.

### 2.4 Intervention

#### 2.4.1 Implementation of intervention

**Figure 2:**
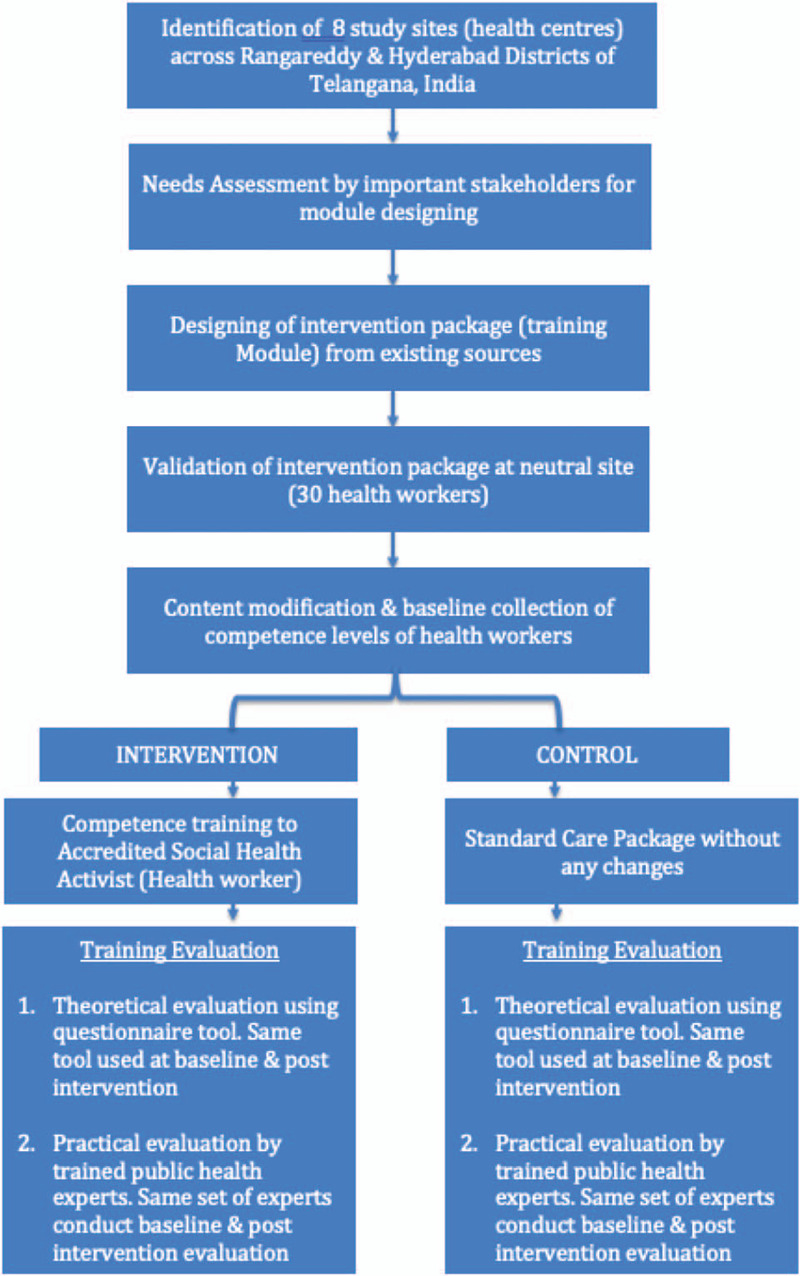
Overview of the intervention.

The implementation of the intervention package consists of training of health workers and evaluation of the training on the clinical competence of the health workers undergoing the training. The intervention will be implemented in the following manner:

1. Identification & Selection of health centers
2. Needs assessment of important stakeholders to improve the efficacy of the subsequent training
3. Development of Intervention package and its validation
4. Identification & recruitment of health workers for study
5. Delivery of Intervention package:
  a. Health worker Training: The first part of the package will focus on the training of the health workers in the intervention arm.
  b. Evaluation of training: The second part of the package will focus on the evaluation of the training. This will be done using the questionnaire tool developed for pre and post-session and pre and post-intervention evaluation of the theoretical competence. An additional practical evaluation will be conducted by a neutral doctor to assess that change in clinical competence. The evaluation mechanism will be identical in both the control and the intervention arms (Fig. 2 showcases the overview of the intervention).

#### 2.4.2 Training of health workers

The HWs in the intervention arm will then undergo the customized training. The training module will include the following topics^[3,28]^:

- NCD's and their prevalence in rural areas
- Importance of prevention, early detection and management
- Diabetes and its prevalence in India and rural India
- Diabetes Pathogenesis,
- Signs and symptoms and basic pathology of diabetes
- Common drugs used, Insulin,
- Importance of self-management,
- Key Skills- Using risk assessment tools, generating awareness history taking, behavior counseling
- Screening, Measurement of blood glucose (using glucometer)
- Complications-
  ∘ Hypoglycemia
  ∘ Wound and ulcers in diabetes
  ∘ Oral complication of diabetes
  ∘ Diabetic Foot
  ∘ Diabetic Nephropathy
  ∘ Diabetic Retinopathy, etc.
- Follow-up & its importance: Motivation for follow-up, Motivation & Support for lifestyle changes, community meetings, patient support groups, continuity of care
- Indian Health System and approach to primary care.

The module will be collated using the available modules which were developed and published by the Ministry of Health and Family Welfare, Government of India, Indian Council of Medical Research operational guidelines, The National Program for Prevention and Control of Cancer, Diabetes, Cardiovascular diseases and Stroke operational guidelines and literature from Indian Institute of Public Health-Hyderabad & Public Health Foundation of India.

After the 6-month training phase, an evaluation will occur. The evaluation will consist of theoretical and practical components which will be conducted using the developed questionnaire and the same set of trained public health experts respectively. The same tools would also be used for baseline collection. Both of these techniques will help assess the change in effective knowledge as well the practical skills about type 2 diabetes mellitus & associated complications. The detailed training plan is described below:

a. The trainers will teach the health workers about type 2 diabetes mellitus, its screening, complications and their screening. The trainers will be chosen based on prior knowledge of type 2 diabetes mellitus and prior teaching experience of evidence-based medicine in community health programs. Their willingness to be facilitators will also be an important factor for their selection.
b. The trainers will participate in a 2-hour course^[36]^ focusing on type 2 diabetes mellitus and its complications. Two experienced faculty with extensive teaching experience will bear responsibility for the same. This training aims to provide the facilitators with sufficient skills and tools to conduct the training meetings successfully.
c. The trainers will be provided with a detailed curriculum to be used in all meetings.
d. An additional 2.5-hour session for trainers will be held three months into the academic detailing phase (i.e., after 3 meetings). This will support trainers during this phase of the intervention. They will also receive an ongoing email and phone support.
e. The trainers will then impart training across a 6-month, 20-hour training to the health workers in the intervention group.
f. The intervention will attempt to facilitate a change in the clinical competence of the health worker undergoing training.

#### 2.4.3 Evaluation

The evaluation of the intervention package will help us understand the change in the competence of health workers in relation to type 2 diabetes mellitus and its complications. It will be conducted in the following manner for both the intervention & the control groups:

Paper based
  Overall pre and post-test questionnaire, which will be derived from the diabetes knowledge test 2 of the Michigan University,^[33,34]^ the inputs from the physician/endocrinologist stakeholders, the assessment tools used by government training modules and evaluation tools developed by PHFI for diabetes training. This will help us understand the change in effective knowledge in the health workers in terms of type 2 diabetes mellitus, screening and complications.
Practical Based
  This will be a practical test based on the training. It will help understand how effectively the training will be implemented by the health workers, when they are in contact with the diagnosed and undiagnosed patients. The scores of this assessment will be compared with the practical assessment carried out at the baseline.

Both the baseline and post training practical evaluation will be conducted by the same set of trained public health experts.

A combination of both paper-based and practical score will elucidate the change in the competence of the health workers.

Both the evaluation methods will be assessed according to objective parameters. It will ensure a comprehensive evaluation of all the components on which training will be provided (Table 1).

**Table 1:**
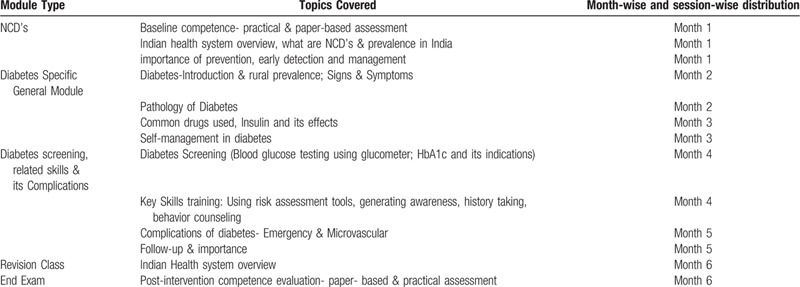
A description of the training schedule of health workers in the intervention group^[28]^.

### 2.5 Data collection, management & analysis

All the data will be collected from both the arms at pre-specified intervals. Pre and post- intervention data will be collected from the control arm. In the intervention arm, pre and post- training data will be collected, at the baseline and after the intervention is concluded. Hence evaluation will be conducted across both the groups at identical time points to obtain an accurate comparison of the training imparted. Qualitative and quantitative data will be collected during and after the intervention. The collected data will be coded and recorded in Microsoft Excel version 2020 (MS Excel). We will use MS Excel for summarizing the descriptive statistics.

These will be obtained from the socio-demographic characteristics, socioeconomic characteristics, lifestyle characteristics, type 2 diabetes screening, health center visits for physician follow-up and health center visits for medicine refill. We will use STATA for data analysis. The principal analysis strategy will be the use of linear mixed models using the R statistical package for computing. This approach provides a simple method to incorporate baseline values and the correlation of each participant over time (using a random effect). This model is more potent than the repeated measures analysis of variance. It can easily examine differences between the trial groups at all time points and can accommodate potential confounders. Subsequent adjustments will be made for potential confounding variables as necessary, such as baseline parameters that may vary between groups.

Data on the confounding factors including sex, literacy, distance from the health centre, marital status will be collected at baseline. Other socio-demographic details, socioeconomic status, and occupation will also be recorded at baseline.

Secondary contrasts will be used to examine differences from baseline for all other time points. However, these will be of exploratory interest only.

### 2.6 Data safety and monitoring

The data will be kept safe with the principal investigator (PI) and later with the data manager on a separate hard disk and local server (password protector) with the access to the same being provided to stakeholders only on a need to share basis.

### 2.7 Data confidentiality

All the information collected will be stored only with the investigators and the data manager. The publications will not reveal any private information of any of the participants. If any qualitative aspect is added at a later stage (like a case study), it will only be done with the participant's consent.

As of now we are not planning on collecting any confidential data which might be harmful for the participants.

## 3 Discussion

This trial will evaluate the use of health worker training as a tool for improving the clinical competence in relation to type 2 diabetes mellitus. We anticipate that the module will provide a greater understanding of type 2 diabetes mellitus, the importance of screening of both disease and complications and improved skills for the same.

Though many studies have focused on health worker training in the high-income nations, there is a dearth of evidence about a competence focused training package for health workers in type 2 diabetes mellitus in low- and middle-income countries like India. The findings of the study can serve as a cornerstone for training policies for health workers. It may help in reducing the cost burden of type 2 diabetes mellitus management on the public sector and the persons living with diabetes mellitus but more research on the impact of such a study will be needed to be undertaken for realizing the same. The results of the study will be disseminated by publishing them in peer reviewed journals.

### 3.1 Strengths

The principal merit of the study is that it is one of a kind. It will help develop a standardized training and evaluation, competence package for health workers, specific to type 2 diabetes mellitus. Very few studies have focused on the same.

### 3.2 Limitations

The study is limited to one district of the country. Hence, local factors contributing to the study's success or failures might not be replicable in all states. Objective measurements are time and resource-consuming and hence subjective practical evaluations are being incorporated.

## Acknowledgments

The authors acknowledge the support provided by researchers from Pushpagiri Vitreoretinal Institute, namely: R. Govindhari, Dr Vijaykiran & Dr Bala Vidyadhar MS for their administrative support in obtaining necessary permissions for the sites of the study.

## Author contributions

**Conceptualization:** Anirudh Gaurang Gudlavalleti, GR Babu, OCP van Schayck, NC Schaper, GVS Murthy.

**Data curation:** Anirudh Gaurang Gudlavalleti, Melissa Glenda Lewis.

**Funding acquisition:** Anirudh Gaurang Gudlavalleti.

**Methodology:** Anirudh Gaurang Gudlavalleti, GR Babu, OCP van Schayck, NC Schaper, GVS Murthy.

**Project administration:** Anirudh Gaurang Gudlavalleti.

**Resources:** Anirudh Gaurang Gudlavalleti, GR Babu, OCP van Schayck.

**Software:** Anirudh Gaurang Gudlavalleti, Melissa Glenda Lewis.

**Supervision:** GR Babu, OCP van Schayck, NC Schaper, GVS Murthy.

**Validation:** GR Babu, O.C.P van Schyack.

**Writing – original draft:** Anirudh Gaurang Gudlavalleti.

**Writing – review & editing:** Anirudh Gaurang Gudlavalleti, GR Babu, OCP van Schayck, NC Schaper, GVS Murthy.

## Supplementary Material

Supplemental Digital Content

## Supplementary Material

Supplemental Digital Content

## References

[1] Institute for Health Metrics and Evaluation (IHME). Findings from the Global Burden of Disease Study 2017. Seattle, WA: IHME, 2018.

[2] IDF. IDF Diabetes Atlas 2017. 2017. doi: http://dx.doi. org/10.1016/S0140-6736(16)31679-8.

[3] Indian Council of Medical Research. ICMR Guidelines for Management of Type 2 Diabetes 2018. New Delhi: Indian Council of Medical Research; 2018. https://main.icmr.nic.in/sites/default/files/guidelines/ICMR_GuidelinesType2diabetes2018_0.pdf

[4] The World Bank. Rural Population (% total population)-India. 2019. https://data.worldbank.org/indicator/sp.rur.totl.zs. Accessed 2 Feb 2019

[5] Abdel-All M Thrift AG Riddell M Evaluation of a training program of hypertension for accredited social health activists (ASHA) in rural India. BMC Health Serv Res 2018;18:1–1. 29720161 10.1186/s12913-018-3140-8 PMC5932780

[6] Kumar V Singh P Access to healthcare among the Empowered Action Group (Eag) states of India: Current status and impeding factors. Natl Med J India 2016;29:267–73. 28098080

[7] Baru RV Acharya A Acharya S Inequities in Access to Health Services in India. Econ Polit Wkly 2010;38:49–58.

[8] Jacobs B Ir P Bigdeli M Addressing access barriers to health services: An analytical framework for selectingappropriate interventions in low-income Asian countries. Health Policy Plan 2012;27:288–300. 21565939 10.1093/heapol/czr038

[9] Jeyashree K Prinja S Kumar MI Inequity in access to inpatient healthcare services for non-communicable diseases in India and the role of out-of-pocket payments. Natl Med J India 2017;30:249–54. 29916423 10.4103/0970-258X.234390

[10] Kanuganti S Sarkar AK Singh AP Quantification of accessibility to health facilities in rural areas. Case Stud Transp Policy 2015;3:311–20.

[11] Anjana RM Deepa M Pradeepa R Prevalence of diabetes and prediabetes in 15 states of India: results from the ICMR–INDIAB population-based cross-sectional study. Lancet Diabetes Endocrinol 2017;5:585–96. 28601585 10.1016/S2213-8587(17)30174-2

[12] Jeet G Thakur JS Prinja S Community health workers for non- communicable diseases prevention and control in developing countries: Evidence and implications. PLoS One 2017;31:1497. 10.1371/journal.pone.0180640 PMC5509237 28704405

[13] Warsi S Elsey H Boeckmann M Using behaviour change theory to train health workers on tobacco cessation support for tuberculosis patients: A mixed-methods study in Bangladesh, Nepal and Pakistan. BMC Health Serv Res 2019;19:1–4. 30683087 10.1186/s12913-019-3909-4 PMC6347762

[14] Alaofè H Asaolu I Ehiri J Community health workers in diabetes prevention and management in developing countries. Ann Glob Heal 2017;83:661–75. 10.1016/j.aogh.2017.10.009 29221543

[15] Anand TN Joseph LM Geetha AV Task sharing with non-physician health-care workers for management of blood pressure in low-income and middle-income countries: a systematic review and meta-analysis. Lancet Glob Heal 2019;7:e761–71. 10.1016/S2214-109X(19)30077-4 PMC6527522 31097278

[16] World Medical Association. WMA resolution on task shifting from the medical profession. J Interprof Care 2009 26–32.

[17] Kamath DY Xavier D Gupta R Rationale and design of a randomized controlled trial evaluating community health worker–based interventions for the secondary prevention of acute coronary syndromes in India (SPREAD). Am Heart J 2014;168:690–7. 25440797 10.1016/j.ahj.2014.07.029 PMC4254408

[18] Srinivasapura Venkateshmurthy N Ajay VS Mohan S m-Power Heart Project - a nurse care coordinator led, mHealth enabled intervention to improve the management of hypertension in India: Study protocol for a cluster randomized trial. Trials 2018;19:1–9. 30086778 10.1186/s13063-018-2813-2 PMC6081824

[19] Mohan S Jarhyan P Ghosh S UDAY: A comprehensive diabetes and hypertension prevention and management program in India. BMJ Open 2018;10:1–0. 10.1136/bmjopen-2017-015919 PMC6082491 29991625

[20] Colleran K Harding E Kipp B Building Capacity to Reduce Disparities in Diabetes: Training Community Health Workers Using an Integrated Distance Learning Model. Diabetes Educ 2012;38:386–96. 22491397 10.1177/0145721712441523

[21] Joshi R Alim M Kengne AP Task shifting for non-communicable disease management in low and middle income countries - A systematic review. PLoS One 2014;9:1–9. 10.1371/journal.pone.0103754 PMC4133198 25121789

[22] Labhardt ND Balo JR Ndam M Task shifting to non-physician clinicians for integrated management of hypertension and diabetes in rural Cameroon: A programme assessment at two years. BMC Health Serv Res 2010;10:339. 21144064 10.1186/1472-6963-10-339 PMC3018451

[23] Lekoubou A Awah P Fezeu L Hypertension, diabetes mellitus and task shifting in their management in sub-Saharan Africa. Int J Environ Res Public Health 2010;7:353–63. 20616978 10.3390/ijerph7020353 PMC2872286

[24] Khetan A Purushothaman R Zullo M Rationale and design of a cluster-randomized controlled trial to evaluate the effects of a community health worker–based program for cardiovascular risk factor control in India. Am Heart J 2017;185:161–72. 28267470 10.1016/j.ahj.2016.10.027

[25] Khatri GR Frieden TR Controlling Tuberculosis in India. N Engl J Med 2002;347:1420–5. 12409545 10.1056/NEJMsa020098

[26] Patel AR Nowalk MP Expanding immunization coverage in rural India: a review of evidence for the role of community health workers. Vaccine 2010;28:604–13. 19896450 10.1016/j.vaccine.2009.10.108

[27] NHM, NHSRC. ASHA Which Way Forward…? New Delhi: Macrographics; 2011.

[28] National Health Mission. Operational Guidelines: Prevention, Screening and Control of Common Non-Communicable Diseases: Hypertension, Diabetes and Common Cancers (Oral, Breast, Cervix)(Part of Comprehensive Primary Health Care. National Health Mission; 2016. http://nhsrcindia.org/sites/default/files/Operational Guideline Comprehensive Primary Health Care.pdf.

[29] Nursing and Midwifery Board of Australia. MIDWIFE STANDARDS FOR PRACTICE. 2018. https://www.nursingmidwiferyboard.gov.au/Codes-Guidelines-Statements/Professional-standards/Midwife-standards-for-practice.aspx.

[30] NHM, NPCDCS. Training manual for NCD program manager at State and District level. 2017.

[31] National Health Mission Ministry of Health and Family Welfare Government of India Module for ASHA on Non-Communicable Diseases 2015.

[32] ASHA Flipbook, India PHF of, Hyderabad II of PH, Medicine LS of H& T, Queen Elizabeth Diamond Jubillee Trust. 2018.

[33] Fitzgerald JT Funnell MM Anderson RM Validation of the Revised Brief Diabetes Knowledge Test (DKT2). Diabetes Educ 2016;42:178–87. 26769757 10.1177/0145721715624968

[34] Collins GS Mughal S Barnett AH Modification and validation of the Revised Diabetes Knowledge Scale. Diabet Med 2011;28:306–10. 21309839 10.1111/j.1464-5491.2010.03190.x

[35] Government of India. Role in Prevention & Control of Non Communicable Diseases (NCDs) 2009.

[36] Aponte J Diabetes Training for Community Health Workers. J Community Med Health Educ 2015 05. 10.4172/2161-0711.1000378 PMC4837952 27110434

