# Supplementary material for: Evaluation of competence training for the minimally trained health worker in type 2 diabetes: A cluster randomized controlled trial
Source: Medicine (Baltimore). 2020 Oct 30;99(44):e22959. doi: 10.1097/MD.0000000000022959 (PMC7598789; doi:10.1097/MD.0000000000022959)
Supplement: Supplemental Digital Content [file medi-99-e22959-s001.docx]

**Appendix 1: Participant Information Sheet**

**Participant Information sheet**

**Evaluation of Competence training of minimally trained health workers in type 2 diabetes mellitus**

**Why are you doing this study?**

The investigators of the study want to assess the effectiveness of a training module to improve competency of the ASHA workers in relation to type 2 diabetes mellitus.

**What will I do if I choose to be in this study?**

You will to take part in a six-month, type 2 diabetes mellitus training and its subsequent evaluation at the health centre. You will have to be regular for the training and compulsorily attend the evaluation of the training (paper based and practical) while also being present for the baseline assessment. You will also need to provide your demographic details like name, age, gender, educational qualification, address, etc. We will not be collecting any confidential information.

**Study duration:** Study participation will take approximately 6-8 months.

**What are the possible risks or discomforts?**

We do not anticipate any risk in your participation other than the possibility that you will have to travel to the health centre and answer a few questions based on the training. You do not have to answer any question that you are not comfortable with and are free to stop at any time. As with all research, there is a chance that confidentiality of the information we collect from you could be breached – we will take steps to minimize this risk.

**What are the possible benefits for me or others?**

Your knowledge and skills in relation to type 2 diabetes mellitus will improve. It will help improve your professional standing in the community. The aftermath of the study may also help us to identify general wellbeing of the patients with T2DM.

**Financial Information**

Participation in this study will involve no direct cost to you apart from travelling to the health centres. You will not be paid for participating in this study.

**What are my rights as a research participant?**

Participation in this study is voluntary. You do not have to answer any question you do not want to answer. If at any time and for any reason, you would prefer not to participate in this study, please feel free not to. You may withdraw from this study at any time, and you will not be penalized in any way for deciding to stop participation.
