# Supplementary material for: Evaluation of competence training for the minimally trained health worker in type 2 diabetes: A cluster randomized controlled trial
Source: Medicine (Baltimore). 2020 Oct 30;99(44):e22959. doi: 10.1097/MD.0000000000022959 (PMC7598789; doi:10.1097/MD.0000000000022959)
Supplement: Supplemental Digital Content [file medi-99-e22959-s002.docx]

**Appendix 2: Potential Study Sites identified in Hyderabad & Rangareddy Districts of Telangana, India**

| S.No | Name of Health Centre | Address of centre |
| --- | --- | --- |
| 1 | Basti Dawakhana Allaudin Koti | Sanathnagar zone; Hyderabad district; Hyderabad; Telangana |
| 2 | Basti Dawakhana Chintabavi | Mettuguda zone; Hyderabad District; Hyderabad; Telangana |
| 3 | Basti Dawakhana Ganganagar | Harajpenta (Ambarpet) Zone; Hyderabad District; Hyderabad; Telangana |
| 4 | Basti Dawakhana Mutyalambagh | Hyderguda, Gaganmahal zone, Hyderabad District; Hyderabad; Telangana |
| 5 | Basti Dawakhana Narsimhabasti | Tilaknagar zone; Hyderabad District; Hyderabad; Telangana |
| 6 | Primary Health Centre Abdullapurmet | Abdullapurmet village; Rangareddy District; Telangana |
| 7 | Primary Health Centre Balapur | Meerpet zone; Hyderabad District; Hyderabad; Telangana |
| 8 | Primary Health Centre Saroornagar | Sri Venkateshwara Colony, Saroornagar; Hyderabad District; Hyderabad; Telangana |
| 9 | Primary Health Centre Serilingampally | Adarsh Nagar, Serilingampally; Rangareddy District; Telangana |
| 10 | Primary Health Centre Sivarampalli | Aramgarh Side, Sivarampalli Village; Hyderabad District; Hyderabad; Telangana |
